# Pharmaceutical expenditure and gross domestic product: Evidence of simultaneous effects using a two‐step instrumental variables strategy

**DOI:** 10.1002/hec.3832

**Published:** 2018-10-10

**Authors:** Mujaheed Shaikh, Afschin Gandjour

**Affiliations:** ^1^ Economics Department Frankfurt School of Finance and Management Frankfurt Germany; ^2^ Health Economics and Policy Vienna University of Economics and Business Vienna Austria

**Keywords:** GDP per capita, income elasticity, instrumental variables, public pharmaceutical expenditure

## Abstract

This paper estimates the income elasticity of government pharmaceutical spending and assesses the simultaneous effect of such spending on gross domestic product (GDP). Using a panel dataset for 136 countries from 1995 to 2006, we employ a two‐step instrumental variable procedure where we first estimate the effect of GDP on public pharmaceutical expenditure using tourist receipts as an instrument for GDP. In the second step, we construct an adjusted pharmaceutical expenditure series where the response of public pharmaceutical expenditure to GDP is partialled out and use this endogeneity adjusted series as an instrument for pharmaceutical expenditure. Our estimations show that GDP has a strong positive impact on pharmaceutical spending with elasticity in excess of unity in countries with low spending on pharmaceuticals and countries with large economic freedom. In the second step, we find that when the quantitatively large reverse effect of GDP is accounted for, public pharmaceutical spending has a negative effect on GDP per capita particularly in countries with limited economic freedom.

## 1 INTRODUCTION

This paper confronts two questions related to medical spending. First, the paper estimates the income elasticity of government pharmaceutical spending, and second, it assesses the simultaneous effect of such spending on income itself. Both questions are important from a health economics and a public spending perspective. The rise in health expenditures in the latter half of the 20th century has increased fiscal constraints for governments worldwide. Policy makers therefore are keen on understanding the determinants of health expenditure to identify the right policy levers that can address budgetary concerns. Concurrently, the understanding of the impact of such spending on economic growth itself is limited. The effect of government spending on economic growth has been a central issue in the growth literature for more than 30 years (Aschauer, 1989; Barro, 1990; Landau, 1983). It is widely recognized that increasing or decreasing government expenditures should be informed on the basis of the contribution of each spending component to economic growth (Devarajan, Swaroop, & Zou, 1996).

There is a voluminous literature that tackles both these issues, yet these questions remain open for further research for several reasons. First, findings from previous literature remain inconclusive concerning the magnitude of the income elasticity of health spending. Some studies argue that health expenditure is a luxury good (Kleiman, 1974; Leu, 1986; Newhouse, 1977) whereas others find it to be a necessity good (Baltagi & Moscone, 2010; Di Matteo, 2003; Farag et al., 2012; Moscone & Tosetti, 2010; Parkin, McGuire, & Yule, 1987). Though findings from past literature thus disagree with respect to the magnitude of the relationship, they concur concerning the sign of the coefficients in that national income relates positively with health expenditure (see, among others, Barros, 1998; Hauck & Zhang, 2016; Hitiris & Posnett, 1992; Getzen, 2000; Xu, Saksena, & Holly, 2011).

The lack of consensus in the elasticities can be attributed to both theoretical and methodological differences. On the theoretical front, micro econometric analyses of health expenditures rely on standard demand theory and find elasticities less than unity. Macro level analyses use models similar in theory to those used by micro studies but find elasticities greater than unity (Gerdtham & Jönsson, 2000). Applying standard demand theory to aggregated data may therefore not be suitable because a nation's income constraint may be more binding than that of an individual, particularly in countries where the state is largely responsible for health care costs (Gerdtham & Jönsson, 2000; Moscone & Tosetti, 2010). On the methodological front, the incongruence in the estimates from different studies arises due to functional form differences in the econometric models, use of different conversion metrics for expenditure and income data, and unaccounted heterogeneity (see,e.g., Getzen, 2000; Gerdtham & Jönsson, 1991; Gerdtham & Jönsson, 1992; Parkin et al., 1987). Further, first generation studies largely relied on cross sections of countries whereas second‐generation studies employed more rigorous econometric methods such as panel data analyses, thereby controlling for several sources of endogeneity (see Gerdtham & Jönsson, 2000, for a detailed overview of these). Despite the research efforts, it is therefore still an open debate whether health care is a luxury good or a necessity good. With regard to The Organisation for Economic Co‐operation and Development (OECD) countries, some papers report that income elasticity is larger than unity, that is, health care is a luxury good (e.g., Gerdtham & Löthgren, 2002; Okunade & Murthy, 2002), whereas others state that the elasticity is less than unity, that is, health care is a necessity good (e.g., Baltagi & Moscone, 2010; Bilgel & Tran, 2013; Dreger & Reimers, 2005; Freeman, 2003). In studies on developing countries, the results also diverge. For example, Bhat and Jain (2004), Jaunky and Khadaroo (2008), and Mehrara and Musai (2011) report income elasticities larger than one, whereas Sahn (1992) among others provides evidence that the income elasticity is less than one. Owing to the differential findings, the income elasticity of health spending remains an open question.

Moreover, past literature has largely focused on total health expenditures, ignoring the different components it comprises. It seems plausible to presume that the elasticity of health spending will depend on the very component of spending being considered. As such, the source of variation in health expenditure matters for the income elasticity of expenditures (Acemoglu, Finkelstein, & Notowidigdo, 2013). To the best of our knowledge, there is no country level analysis that explores the income elasticity of public pharmaceutical expenditures in both high‐ and low‐income countries. 1Acemoglu et al. (2013) assess the response of hospital expenditures to income and further break it down into payroll expenditure, employee expenditures, skill composition, hospital utilization, etc. This seems surprising because there are several reasons why this type of spending should be of particular interest. First, it forms a large part of total health expenditure and has increased by almost 50% since 2000 (OECD, 2011), which has exposed it to several regulations and cost containment measures. Second, policy makers are endorsing the view that savings can be achieved by regulating pharmaceutical markets and reducing costs without affecting the quality of care (Carone, Schwierz, & Xavier, 2012). Finally, income elasticity of pharmaceutical spending has important implications in terms of pharmaceutical policy itself.

With respect to the second question of our paper, the effect of public pharmaceutical spending on economic growth, the question is of interest from different perspectives just as the answer remains ambiguous from these perspectives. From a health economics perspective, inasmuch as spending on health leads to improved health outcomes, a positive relationship between health spending and economic growth can be expected. Although some papers find a positive link between health and economic growth (Bloom, Canning, & Sevilla, 2004), others find no effect of health on growth (Acemoglu & Johnson, 2007; Ashraf, Lester, & Weil, 2008; Hansen & Lønstrup, 2015). The effect of health spending therefore remains questionable. From a pure public spending perspective, findings from the literature also add to the debatable effects of health spending on gross domestic product (GDP). Some of the previous literature finds a negative relationship between excessive public spending and GDP (Devarajan et al., 1996; Miller & Russek, 1997) whereas others consider health spending a “productive” form of public expenditure that affects growth positively (Bleaney, Gemmell, & Kneller, 2001; Glomm & Ravikumar, 1997). 2We acknowledge that in order to understand the full picture of the response of pharmaceutical spending to GDP growth and vice‐versa, an analysis of total pharmaceutical expenditures and not just the public component of it is essential. This is particularly relevant for low‐income countries where public spending levels are low and private spending is an important method of financing. However, we are limited in our analysis owing to data concerns with respect to private spending that is part of total spending. Certain components are not included in total spending such as medicines covered by private insurance, produced by local pharmacists and hospitals, and informal sales or payments (e.g., unregistered or resale pharmaceuticals). These largely represent private spending in low‐income countries that would be heavily underestimated. Moreover, we consider the question of how private spending on pharmaceuticals affects growth only of relevance if it were an option to contain private spending. Yet, based on the values of a democratic society and its public discourse, citizens shall not be prohibited from purchasing pharmaceuticals in a private market if these pharmaceuticals are not covered by their insurance. That is, based on democratic values strict rationing seems hardly defendable and requires enforcement, rendering the question of whether to contain private spending essentially a hypothetical one.


Therefore, in this paper, we assess the effect of income measured in GDP per capita on public pharmaceutical expenditure per capita and the reverse effect using a sample of 136 countries from 1995 to 2006. We use a relatively recent two‐step instrumental variable (IV) procedure (from the aid effectiveness literature; see Brückner, 2011) to assess the causal effect of GDP on pharmaceutical spending in the first step and then assess the effect of pharmaceutical spending on GDP in the second step after accounting for the simultaneous effect of GDP from the first step. We find that the income elasticity of public pharmaceutical expenditure is greater than unity in the full sample, and that there is a negative effect of public pharmaceutical expenditure on GDP per capita. However, important heterogeneous patterns exist. GDP per capita has a statistically significant positive effect on pharmaceutical spending only for high‐income countries, and pharmaceutical spending has a statistically significant negative effect only for low‐income countries. Quantile regressions show that the income elasticity of pharmaceutical spending varies across the distribution of pharmaceutical spending. Similarly, the negative effect of pharmaceutical expenditure on GDP is statistically significant only for the middle (25th, 50th, and 75th) quantiles of income. Finally, based on the index of Economic Freedom of the World (EFW) by the Fraser Institute, we find that GDP per capita does not have an effect on pharmaceutical expenditure in countries that are classified as “not free.” However, pharmaceutical spending has a statistically significant negative effect on GDP per capita in these countries.

Our paper contributes at several levels. First, it contributes directly to the literature that assesses the effect of public spending on economic growth. Although there is some literature on the effect of total health expenditure, we found no paper specifically analyzing public spending on pharmaceuticals. This paper attempts to fill that gap. Second and more importantly, our contribution to the literature in health economics is twofold by—(a) assessing the income elasticity of public pharmaceutical spending across a multitude of countries using IVs, something which past studies have not considered, 3Farag et al. (2012) and Acemoglu et al. (2013) are notable exceptions that used instrumental variables. However, their dependent variables were different. and (b) assessing the effect of pharmaceutical spending on a nonhealth outcome such as economic growth.

The paper is organized as follows. In Section 2, we discuss the econometric methodology. Section 3 introduces the data and presents descriptive statistics. In Section 4, we present and discuss our findings along with different sensitivity tests. Section 5 revisits instrument validity, and Section 6 presents results of the heterogeneity analysis. Section 7 discusses the effect of pharmaceutical expenditure on GDP, and Section 8 concludes with policy implications.

## 2 ECONOMETRIC FRAMEWORK

To assess the effect of GDP per capita on public pharmaceutical spending per capita and the reverse effect within the same econometric framework, we employ a two‐step IV procedure outlined in Brückner (2011) and Moreno‐Serra and Smith (2015). 4To the best of our knowledge, Moreno‐Serra and Smith (2015) are the only other authors within health economics to have used the two‐step IV procedure. Throughout the remainder of the paper, GDP refers to GDP per capita and pharmaceutical expenditure refers to public pharmaceutical expenditure per capita. In the first step, we assess the causal effect of GDP on pharmaceutical expenditure using an IV approach. In the second step, because we are also interested in estimating the effect of pharmaceutical expenditure on GDP, we generate a new endogeneity adjusted pharmaceutical expenditure series using the residual variation in pharmaceutical expenditure from the first step (where the response of pharmaceutical spending to GDP is partialled out). We describe the two‐step procedure in detail now.

Step 1: Estimating the causal effect of GDP on pharmaceutical expenditure.

Consider the following regression specification:

(1) $$Y_{\mathrm{it}}=\beta X_{\mathrm{it}}+\mu W_{\mathrm{it}}+\alpha_{i}+\gamma_{t}+\varepsilon_{\mathrm{it}},$$

where the subscripts *i* and *t* represent country and time, respectively. *Y*_*it*_ is the dependent variable (public pharmaceutical expenditure per capita) to be explained by *X*_*it*_, which is the variable of main interest (GDP per capita). *W*_*it*_ is a vector of time varying controls that is included in the specification along with a full set of country dummies *α*_*i*_ that capture the effect of time‐invariant unobservables, and a full set of time dummies *γ*_*t*_ aimed at capturing common shocks to the countries. ε_*it*_ represents the error term that captures all sources of endogeneity. 5The choice of a fixed effects model is motivated by the fact that there could be omitted variables (time‐invariant and time varying) that relate to both pharmaceutical expenditure and GDP. For example, geographic location may have a strong impact on disease burden and on economic growth (Boulhol, de Serres, & Molnar, 2008; Gallup, Sachs, & Mellinger, 1999). The assumptions of a random effects model are, therefore, not satisfied. Nevertheless, we report results of the Hausman tests in Table 2, which support fixed‐effects specifications. Although fixed effects may serve as a useful framework to overcome time‐invariant unobserved heterogeneity, two sources of bias remain and therefore Cov(*X*_*it*_, ε_*it*_) ≠ 0. First, time varying unobserved variables (omitted variables) are particularly concerning because these have the potential to upward or downward bias the estimates. Second and more crucial is the reverse causal effect of pharmaceutical expenditure itself on GDP, which can lead to an upward bias in Equation (1) if pharmaceutical expenditure is positively related to GDP. In order to overcome these issues and to appropriately estimate the effect of GDP on pharmaceutical expenditure, we need an instrument *Z*_*it*_ that is correlated strongly with *X*_*it*_ (i.e., GDP; condition of relevance) and is excluded from the second stage (exclusion restriction).

We use international tourist receipts as an instrument for GDP. International tourist receipts reflect expenditures by international inbound visitors. These are, as defined by the World Tourism Organization, receipts earned by the destination country and cover spending on lodging, food and drinks, fuel, transport, entertainment, shopping, etc. The relationship between tourism and the economy is intuitively straightforward. Tourism directly or indirectly generates an increase in economic activity and results in an increase in the wealth of the residents through, for instance, the services they provide and employment. As such, it should be positively related to the GDP of a country. The tourism led growth hypothesis has been extensively tested and supported in the tourism‐growth literature (Brida & Pulina, 2010; Brida, Risso, Lanzilotta, & Lionetti, 2010; Gunduz & Hatemi‐J, 2005; Lean & Tang, 2009; Risso & Brida, 2009). Although the instrument easily satisfies the relevance criteria as shown by the first stage results and F‐statistics that are well beyond the conventional benchmark of 10, we argue for the validity of the IV in what follows.

Our exclusion restriction requires that Cov(*Z*_*it*_, ε_*it*_| *W*_*it*_, *α*_*i*_, *γ*_*t*_) = 0, that is, international tourist receipts be unrelated to public pharmaceutical expenditure except through GDP. We do not fathom why tourist expenditures should be related to public pharmaceutical expenditures or should have a substantial effect on them that should be of concern. However, one possible threat to the instrument is the existence and extent of medical tourism in a country. Even then, we posit that this should at least not be related to “public” pharmaceutical expenditures of a country, because a medical tourist will in general pay for his health care privately. Even if insurance pays for the tourists, it should be reflected in the expenditure of the home country. We also conduct other tests in support of our instrument, which are discussed in Section 6.

Step 2: Estimating the effect of pharmaceutical expenditure on GDP.

Now consider a reverse econometric framework of Equation (1)

(2) $$X_{\mathrm{it}}=\delta Y_{\mathrm{it}}+\mu W_{\mathrm{it}}+\alpha_{i}+\gamma_{t}+\varepsilon_{\mathrm{it}}.$$

If GDP has a significant causal effect on pharmaceutical spending estimated from Step 1, then the ordinary least squares estimate of the effect of pharmaceutical expenditure on GDP will suffer from this endogeneity bias, that is, Cov(*Y*_*it*_, ε_*it*_) ≠ 0. This endogeneity bias however can be taken care of by constructing an adjusted pharmaceutical spending series ( $Y_{\mathrm{it}}^{*}\;$in Equation ((3)) below) where the response of pharmaceutical spending to GDP is partialled out, that is,

(3) $$Y_{\mathrm{it}}^{*}=Y_{\mathrm{it}}-\beta X_{\mathrm{it}}.$$

This endogeneity adjusted pharmaceutical expenditure series is free of the simultaneity bias due to GDP by construction. Using the residual variation in expenditure that is not driven by GDP as an instrument for pharmaceutical expenditure in ((1)) adjusts for the reverse causality and provides a consistent estimate. This procedure assumes that the exclusion restriction in ((1)) holds, that is, error terms in ((1)) and ((2)) are uncorrelated. If there are omitted variables that are part of both Equations (1) and 2, then the correlation between the two error terms is likely. However, the procedure will still solve the reverse causality problem and provide consistent estimates (see Brückner, 2011, for details on the procedure). It is important to mention here that there will be correlation between the error terms of the two equations only if there are omitted variables that will affect the within‐country changes in both GDP and pharmaceutical expenditure simultaneously. For this reason, we also include several time varying controls in *W*_*it*_ that may be important and may determine both GDP and pharmaceutical expenditure along with country and time fixed effects that take into account other unobservable heterogeneity related to country specific characteristics and common shocks, respectively.

## 3 DATA AND DESCRIPTIVE STATISTICS

The data we use in this paper come from different sources. The data on pharmaceutical expenditures come from the World Health Organization's World Medicines Situation 2011—Medicine Expenditures' annex. The data are publicly available at http://apps.who.int/medicinedocs/en/d/Js20052en/ [Last accessed 23 February 2018]. The remainder of the data that we use in our analysis comes either from the World Bank Open Data or from the International Monetary Fund's World Economic Outlook database. The dataset we use is an unbalanced panel from 1995 to 2006 for a total of 136 countries. Therefore, we have around 1,389 observations in total with around 10 observations per country on average. The variables of interest are described in detail in the following.

Public pharmaceutical expenditure per capita comes from the World Health Organization (WHO) and is expressed in U.S. dollars (at exchange rate). Comparability of expenditures between countries has been a key issue in past studies. One of the advantages of this dataset is that it is based on a WHO methodology that promotes comparable country data on health system expenditures. Total pharmaceutical expenditures that provide a measure of consumption of pharmaceuticals are disaggregated to reflect public and private expenditures. The data, however, do not distinguish between generic or branded medicines and are regardless of the means or place of distribution. For a detailed overview of the data, we refer readers to the World Medicines Situation 2011 report. In our analysis sample, average annual public pharmaceutical expenditure per capita is $64 with a maximum of $832. For high‐income countries, it is $108 whereas for low‐income countries it is $5. Table 1 shows the summary statistics for all variables for the overall sample and by income group and Table A1 lists the countries used in the analysis. 6Due to sample size issues, we pool high‐income and upper middle‐income countries into one group called “high‐income” countries and lower and lower‐middle income countries into another group called “low‐income” countries. Later on in the analysis, we use the two‐income groups as one way of assessing heterogeneity.

**Table 1:** Summary statistics

Variable	Full sample		Std. dev.	Min	Max
	Obs.	Mean			
Public pharmaceutical exp. per capita ($US)	1,389	64.12639	106.3668	0.041849	831.9869
GDP per capita ($US)	1,389	13583.8	17704.4	233.1437	103060.5
Remaining health exp. per capita ($US)	1,389	798.5446	937.5408	12.56647	6787.797
Tourist receipts (millions [$US])	1,389	4.76E + 09	1.25E + 10	400000	1.27E + 11
Life expectancy at birth (years)	1,369	70.14461	7.928318	42.1582	82.32195
Gross savings (% of GDP)	1,175	20.94163	10.89711	−146.164	64.71603
Capital formation (% of GDP)	1,309	24.34591	12.91513	0	219.0694
Total government expenditure (% of GDP)	1,289	31.22698	12.44523	9.328	128.289
Population over 65 years (% of total)	1,365	8.537837	4.868295	2.198119	20.39357

	High income				
Variable	Obs.	Mean	Std. dev.	Min	Max
Public pharmaceutical exp. per capita ($US)	797	107.9008	123.2819	1.442033	831.9869
GDP per capita ($US)	797	22307.84	19147.86	981.5044	103060.5
Remaining health exp. per capita ($US)	797	1257.722	1011.799	68.26747	6787.797
Tourist receipts (millions ($US))	797	7.45E + 09	1.58E + 10	400000	1.27E + 11
Life expectancy at birth (years)	777	74.39379	4.87424	50.35488	82.32195
Gross savings (% of GDP)	709	21.95556	11.25445	−146.164	64.71603
Capital formation (% of GDP)	781	25.44211	15.1979	0.298644	219.0694
Total government expenditure (% of GDP)	751	35.91555	11.07614	11.77	128.289
Population over 65 years (% of total)	773	11.19371	4.541596	2.198119	20.39357

	Low income				
Variable	Obs.	Mean	Std. dev.	Min	Max
Public pharmaceutical exp. per capita ($US)	592	5.19356	6.165289	0.041849	42.5232
GDP per capita ($US)	592	1838.763	1229.937	233.1437	5596.732
Remaining health exp. per capita ($US)	592	180.362	135.5475	12.56647	692.0238
Tourist receipts (millions ($US))	592	1.15E + 09	3.34E + 09	800000	3.39E + 10
Life expectancy at birth (years)	592	64.56755	7.707776	42.1582	76.28613
Gross savings (% of GDP)	466	19.39897	10.14878	−14.3849	54.65947
Capital formation (% of GDP)	528	22.72446	8.229088	0	61.90938
Total government expenditure (% of GDP)	538	24.68214	11.24678	9.328	104.389
Population over 65 years (% of total)	592	5.069947	2.546296	2.420117	16.10646

*Note*. GDP: gross domestic product.

GDP per capita data are from the World Bank database and expressed in U.S. dollars based on purchasing power parities. GDP per capita is a common measure of economic growth and has been used extensively in the literature (some examples are Barro, 1991; Barro & McCleary, 2003; Bloom et al., 2004; Grossman & Krueger, 1995). The average GDP per capita in our full sample is $13,584; for high‐income countries, it is $22,308, and for low‐income countries, it is $1,839.

**Figure 1:**
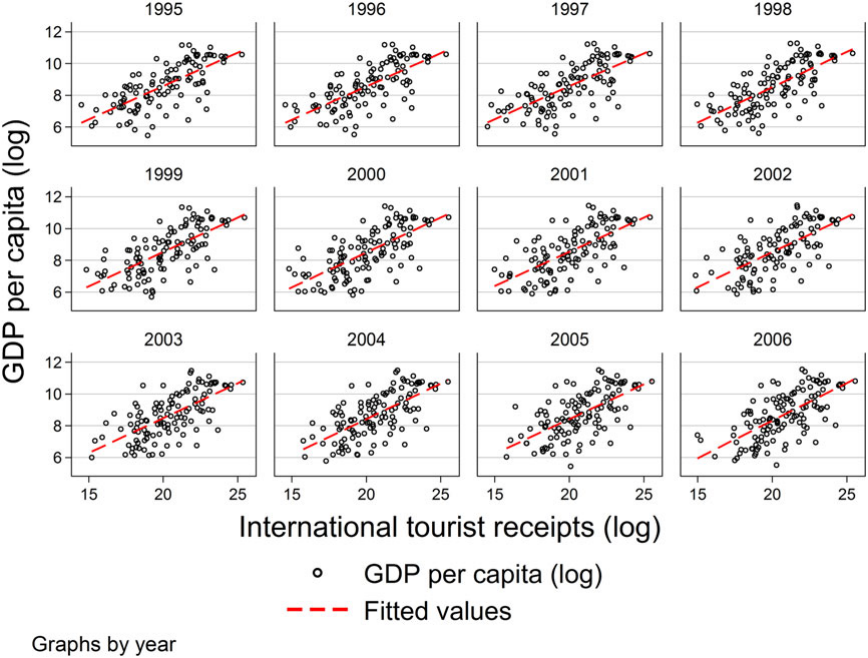
Gross domestic product (GDP) and international tourist receipts [Colour figure can be viewed at wileyonlinelibrary.com]

The instrument, international tourist receipts, also comes from the World Bank database and is expressed in U.S. dollars. As mentioned earlier, international tourism receipts are expenditures by international inbound visitors and were found to have a strong positive relationship with economic growth by previous literature. As seen in the summary statistics in Table 1, income from tourist receipts is much lower for low‐income countries compared with high‐income countries. One can speculate that the contribution of tourist receipts to GDP may be heterogeneous based on income levels, and hence, the first stage relationship between tourist receipts and GDP may be stronger for high‐income than low‐income group of countries. Figure 1 shows the raw correlation of tourist receipts with GDP per capita per year. It can be seen that the correlation between tourism receipts and GDP per capita is positive across all the years of the analysis. Our first stage F‐statistics shown later in the results section are clearly above the thumb rule of 10 for both the income groups and for the overall sample, thus alleviating the concern of a weak first stage relationship for the low income group.

We include a host of other variables in our analysis that could be possible sources of omitted variable bias in the regressions. First, we control for health expenditure other than public pharmaceutical expenditure because it seems likely that this may drive both GDP and public pharmaceutical spending. We also control for life expectancy at birth as a measure of health at the country level. The direction in which health will bias the results is ambiguous a priori because it may be either positively (Bloom et al., 2004; Bloom & Canning, 2000) or negatively (Acemoglu & Johnson, 2007; Ashraf et al., 2008; Hansen & Lønstrup, 2015) related to income. Although we are not interested in the effect of health per se, it is necessary that we show robustness of our results to the inclusion of this variable. We also control for variables that are considered important in the macro literature such as savings rate, demographic structure of the country (population over 65 years of age), gross capital formation, and total government expenditures. Summary statistics for all these variables can also be seen in Table 1. To interpret the estimates as elasticities, we take the log form of both GDP and public pharmaceutical expenditure.

## 4 MAIN RESULTS

### 4.1 Effect of GDP per capita on public pharmaceutical expenditure

Table 2 shows the results of the effect of GDP per capita on public pharmaceutical expenditure per capita. Column (1) presents fixed effects estimates which show a significant positive relationship between GDP and pharmaceutical expenditure. The magnitude of the coefficient indicates that pharmaceutical expenditure has an income elasticity of less than unity, similar to the findings of some of the previous literature with respect to total health expenditures. However, these estimates cannot be considered as reflecting the causal effect of GDP on pharmaceutical spending because these are riddled with endogeneity problems discussed previously. Although the regression includes country and time fixed effects, thereby accounting for fixed sources of heterogeneity, other issues with regard to time varying variables and reverse causality remain. Hence, these estimates should be taken at face value simply for comparison purposes with the IV estimates.

**Table 2:** GDP per capita and public pharmaceutical expenditure per capita

Variables	(1)	(2)	(3)	(4)
	FE OLS	First stage	Reduced form	2SLS
	Pharmaceutical expenditure	GDP per capita (natural log)	Pharmaceutical expenditure	Pharmaceutical expenditure
GDP per capita (natural log)	0.709*** (0.215)			1.406** (0.653)
Tourist receipts		0.0935*** (0.0240)	0.131** (0.0514)	
Year FE	Yes	Yes	Yes	Yes
Country FE	Yes	Yes	Yes	Yes
Observations	1,389	1,389	1,389	1,389
R‐squared	0.476	0.563	0.458	0.445
First stage F‐stat				15.12
*p* value				0.0002
Kleibergen‐Paap LM statistic				9.97
*p* value				0.0016
Hausman test statistic	25.46	29.60	279.908	279.907
*p* value	0.0128	0.0000	0.0000	0.0000
Endogeneity test				2.37
*p* value				0.1232
No. of countries	136	136	136	136

*Note*. The DV in columns (1), (3), and (4) is public pharmaceutical expenditure per capita (natural log), whereas in column (2), it is GDP per capita (natural log). Column (1) shows the fixed effects estimate of the effect of GDP per capita (natural log) on the DV. Column (2) shows the first stage relationship between GDP per capita and the instrument tourist receipts (natural log). Column (3) shows the reduced form effect of the instrument on the DV. Column (4) shows the IV estimate of the effect of GDP on the DV. DV: dependent variable; GDP: gross domestic product; IV: instrumental variable; OLS: ordinary least squares.

*** *p* < 0.01.

** *p* < 0.05.

* *p* < 0.1.

Before presenting the IV estimates, we show the first stage relationship of the instrument and the endogenous variable. Column (2) presents the results of the first stage relationship between tourist receipts and GDP per capita. The regression results confirm the graphical evidence from Figure 1. In line with the “tourism led growth” hypothesis and findings of previous literature (Brida et al., 2010; Brida & Pulina, 2010; Gunduz & Hatemi‐J, 2005; Lean & Tang, 2009; Risso & Brida, 2009), we see that tourism expenditures by international inbound visitors are positively and significantly related to GDP per capita. First stage R‐squared is high, indicating that the instrument fulfills the relevance criteria, and it provides estimates in the direction that one would expect. Next, in column (3), we show the reduced form estimate, that is, the relationship between the instrument and pharmaceutical expenditure. If the reduced form effect is not significantly different from zero, the effect of interest can be presumed to be absent or the instrument too weak (Angrist & Krueger, 2001). Column (3) shows that tourist receipts have a statistically significant positive effect on pharmaceutical expenditure per capita in the reduced form as one would have expected. Finally, in column (4), we present the two‐stage least squares estimate that uses tourist receipts as an instrument for GDP per capita. The first stage F‐statistic is 15.12 (significant at a 1% level), indicating that the instrument is strong enough to predict GDP. The Kleibergen‐Paap LM statistic is also strongly significant. The second stage estimate of GDP per capita on public pharmaceutical expenditure is positive and significant at a 1% level, and the magnitude of the coefficient is much larger than the least squares estimate in column (1). The larger coefficient size could be due to a number of reasons. If there are unobserved variables that are negatively (positively) related to GDP and positively (negatively) related to pharmaceutical spending, a downward bias could be observed. For example, disease burden of a country could lead to an underestimate of the true effect of GDP on pharmaceutical expenditure. 7Note that the endogeneity test suggests that GDP per capita is exogenous; however, results presented in columns (3) and (5) of Table 3 below suggest that it is indeed endogenous. In any case, we have outlined strong theoretical reasons and prior empirical evidence that leaves little contention concerning the endogenous nature of economic growth. Moreover, in Step 2 of the empirical strategy where we assess the impact of pharmaceutical expenditure on GDP, the test strongly suggests pharmaceutical expenditures as endogenous (see Table 4).


Quantitatively, the IV estimate suggests that a 1% increase in GDP per capita increases public pharmaceutical expenditure per capita by 1.4%, implying that public pharmaceutical spending has an elasticity greater than unity and hence is a luxury good. This two‐stage least squares estimate is causal to the extent that the exclusion restriction is satisfied, that is, tourist receipts do not have any systematic relationship with public pharmaceutical expenditure other than through GDP per capita. We further discuss the validity of the instrument later in Section 6.

### 4.2 Robustness tests

To test the sensitivity of our results, we perform different robustness checks. First, issues around sample size and outliers are dealt with. Second, robustness of the point estimates is tested to the inclusion of other covariates that have the potential to confound the relationship between GDP and pharmaceutical expenditure. 8In addition, to avoid generated regressor bias in step 2 of the analysis, i.e. the effect of pharmaceutical expenditure on GDP, we run these regressions with bootstrapped standard errors to which the results remain robust. These results are available from the authors upon request.


Table 3 provides all these robustness checks. In column (1), we run regressions only on observations that produce a balanced panel from 1995 to 2006. The point estimates of column (1) are similar in magnitude to those of column (4) in Table 2. Next, in column (2), we exclude outliers from the balanced panel, identified using the Hadi (1992) procedure. This leads to the exclusion of 363 observations. The point estimates are still positive and statistically significant at a 10% level; however, the magnitude of the coefficient is now slightly higher. Further, in column (3), we include variables deemed important in both the health and growth literature as controls in our models. Specifically, we control for what we consider demand side variables such as health (proxied by life expectancy at birth) and demographic structure of the population by controlling for population over the age of 65 years. On the supply side, we control for health expenditure per capita excluding pharmaceutical expenditure and total government expenditure as a percentage of GDP. Variables considered important in the macro growth literature such as gross savings (as a percentage of GDP) and gross capital formation (as a percentage of GDP) are also included as further control variables. In column (4), we include these control variables along with the exclusion of identified outliers. In both cases, the coefficients are qualitatively similar. The coefficients are significant at a 5% level in both columns (3) and (4) and the magnitudes well above unity.

**Table 3:** Robustness tests

Variables	(1)	(2)	(3)	(4)	(5)
	Balanced panel	Outliers (O)	Controls (C)	O + C	C + L
	Pharmaceutical expenditure	Pharmaceutical expenditure	Pharmaceutical expenditure	Pharmaceutical expenditure	Pharmaceutical expenditure
GDP per capita (natural log)	1.654* (0.989)	2.046* (1.175)	2.566** (1.008)	1.878** (0.853)	1.953** (0.833)
Health expenditure			−0.305 (0.317)	−0.203 (0.255)	−0.273 (0.264)
Life expectancy			−0.621 (2.058)	−0.299 (1.909)	−1.039 (2.074)
Capital formation			−0.00186 (0.00686)	0.00195 (0.00864)	−0.00254 (0.00398)
Savings rate			−0.00931* (0.00477)	−0.00757 (0.00502)	−0.00699 (0.00489)
Govt. expenditure			0.0120*** (0.00392)	0.0143*** (0.00364)	0.00365 (0.00542)
Population over 65			0.177 (0.574)	0.318 (0.494)	−0.136 (0.444)
Lag (1) pharmaceutical exp.					0.573*** (0.0839)
Year FE	Yes	Yes	Yes	Yes	Yes
Country FE	Yes	Yes	Yes	Yes	Yes
Observations	1,152	789	1,063	551	970
R‐squared	0.486	0.325	0.468	0.358	0.657
First stage F‐stat	7.42	5.35	11.03	8.57	10.65
*p* value	0.007	0.023	0.0012	0.004	0.0015
Kleibergen‐Paap LM statistic	6.12	4.60	7.30	5.47	8.22
*p* value	0.0133	0.032	0.0069	0.0193	0.0041
Hausman test statistic	17.328	204.099	77.100	67.704	181.19
*p* value	0.0000	0.0000	0.0000	0.0000	0.0000
Endogeneity test	1.58	2.52	2.86	2.09	4.21
*p* value	0.2083	0.1119	0.0905	0.1476	0.0401
Number of countries	96	72	120	73	110

*Note*. The DV in all columns is public pharmaceutical expenditure per capita. All estimates are IV estimates. Column (1) shows estimates using a balanced sample; column (2) shows estimates after excluding outliers from the balanced panel identified using the Hadi procedure; column (3) shows estimates after inclusion of relevant controls in the full sample; column (4) shows estimates after inclusion of controls and exclusion of outliers from the full sample; column (5) shows estimates from a dynamic model that includes the first lag of pharmaceutical expenditure and thereby accounts for inertia in pharmaceutical spending. Health expenditure here refers to all other health spending excluding public pharmaceutical spending. DV: dependent variable; GDP: gross domestic product; IV: instrumental variable.

*** *p* < 0.01.

** *p* < 0.05.

* *p* < 0.1.

Finally, an important issue, also raised in prior analyses, relates to health expenditure inertia. It seems reasonable to assume that current pharmaceutical spending depends on past spending behavior. Therefore, it becomes important to assess robustness by accounting for this inertia in pharmaceutical expenditure. We run a dynamic model including the lags of pharmaceutical expenditure in the regressions where we follow prior literature concerning the choice of the number of lags. Okunade and Suraratdecha (2000) consider one lag as appropriate, whereas Moreno‐Serra and Smith (2015) use up to two lags. For brevity, we show the results of the dynamic model in column (5) of Table 3 using only one lag; however, we have also tested robustness using two lags of expenditure. The results are similar.

Overall, the point estimates are always above unity implying that pharmaceutical spending is a luxury good, which is in contrast to findings from some of the recent studies that use panel data and assess the income elasticity of total health expenditures (Baltagi & Moscone, 2010; Di Matteo, 2003; Farag et al., 2012; Freeman, 2003; Sen, 2005). Our results therefore point out that different components of health spending may behave differently and exhibit different elasticity estimates. Therefore, policy makers should consider this heterogeneity before introducing measures targeting health expenditures.

### 4.3 Effect of public pharmaceutical expenditure on GDP per capita

Our second research question aims at assessing the effect of public pharmaceutical spending on economic growth. We present the results here. The analysis so far suggests that public pharmaceutical expenditure is highly endogenous to GDP per capita. To be precise, the IV estimates suggest that per capita GDP has a strong positive effect on public pharmaceutical spending per capita, which is both economically and statistically significant. Therefore, an ordinary least squares estimation of the effect of public pharmaceutical spending on GDP will most likely be upward biased if the reverse positive effect of per capita GDP on pharmaceutical expenditure is not addressed. While controlling for unobserved heterogeneity in the form of fixed effects and several other confounders may solve the problem of omitted variable bias to some extent, the bias due to reverse causality will still persist. We therefore run our regressions as mentioned in Step 2 of the econometric framework (outlined in Section 2) where the residual variation in public pharmaceutical expenditure is used as an instrument for public pharmaceutical expenditure.

Table 4 presents results of the effect of public pharmaceutical expenditure per capita on GDP per capita. Column (1) shows IV estimates using the full sample. We find that public pharmaceutical spending is negatively related to GDP per capita. The estimate is significant at a 5% level and quantitatively means that a 1% increase in public pharmaceutical expenditure is related to a 0.09% decrease in GDP per capita. Although this result seems counter‐intuitive to what most health economics literature would presume, 9To the extent that health expenditures improve health, and health improves productivity, health expenditures should be positively related to economic growth (Bhargava, Jamison, Lau, & Murray, 2001; Bloom et al., 2004; Bloom & Canning, 2000). A recent study examining the direct link between public health spending and economic growth has found a positive relationship between the two (Halıcı‐Tülüce, Doğan, & Dumrul, 2016). it is in line with some of the other literature that assesses the effect of government spending on economic growth (Devarajan et al., 1996; Miller & Russek, 1997). Next, we assess the robustness of the results in a similar way as we did in Table 3. Analyzing only a balanced sample of countries, excluding outliers using the Hadi (1992) procedure, and including further control variables, we find the same result. In each of these tests, public pharmaceutical expenditure is negatively related to GDP per capita.

**Table 4:** Public pharmaceutical expenditure per capita and GDP per capita

Variables	(1)	(2)	(3)	(4)	(5)	(6)
	Overall	Balanced panel	Outliers (O)	Controls (C)	O + C	C + L
	GDP per capita (natural log)	GDP per capita (natural log)	GDP per capita (natural log)	GDP per capita (natural log)	GDP per capita (natural log)	GDP per capita (natural log)
Pharmaceutical exp.	−0.0915** (0.0400)	−0.0978*** (0.0363)	−0.158*** (0.0526)	−0.0842*** (0.0242)	−0.071*** (0.0239)	−0.112*** (0.0313)
Health expenditure				0.244*** (0.0542)	0.166** (0.0688)	0.260*** (0.0470)
Life expectancy				−0.712 (0.498)	−0.743 (0.585)	−0.251 (0.566)
Capital formation				0.00444*** (0.00103)	0.00490*** (0.00157)	0.00251* (0.00138)
Savings rate				0.00176 (0.00139)	0.00351* (0.00173)	0.00296* (0.00168)
Govt. expenditure				−0.000201 (0.00119)	−0.000728 (0.001645)	0.000493 (0.00186)
Population over 65				0.328** (0.135)	0.336 (0.221)	0.311*** (0.118)
Lag (1) pharmaceutical exp.						0.108*** (0.0322)
Year FE	Yes	Yes	Yes	Yes	Yes	Yes
Country FE	Yes	Yes	Yes	Yes	Yes	Yes
Observations	1,389	1,152	789	1,063	551	970
R‐squared	0.479	0.564	0.443	0.655	0.603	0.657
First stage F‐stat	395.2	366.1	146.6	372.7	605.2	385.63
*p* value	0.000	0.000	0.000	0.000	0.000	0.000
Kleibergen‐Paap LM statistic	32.14	24.26	15.94	22.00	12.45	14.47
*p* value	0.0000	0.0000	0.0001	0.0000	0.0004	0.0001
Endogeneity test	10.77	20.06	16.43	27.98	17.35	29.56
*p* value	0.0010	0.0000	0.0001	0.0000	0.0000	0.0000
No. of countries	136	96	72	120	73	110

*Note*. The DV in all columns is GDP per capita (natural log). All estimates are IV estimates. Column (1) shows estimates of the effect of public pharmaceutical expenditure per capita on GDP per capita (natural log) for the full sample. Column (2) shows estimates using a balanced panel; column (3) shows IV estimates after excluding outliers from the balanced panel identified using the Hadi procedure; column (4) shows estimates after inclusion of relevant controls in the full sample; column (5) shows estimates after inclusion of controls and exclusion of outliers from the full sample; column (6) shows estimates from a dynamic model that includes the first lag of pharmaceutical expenditure and thereby accounts for inertia in pharmaceutical spending. Health expenditure here refers to all other health spending excluding public pharmaceutical spending. DV: dependent variable; GDP: gross domestic product; IV: instrumental variable.

*** *p* < 0.01.

** *p* < 0.05.

* *p* < 0.1.

### 4.4 Intertemporal effects

Given the typical short term thinking of policy makers, we have so far focused on an analysis of concomitant effects. However, it is plausible that the relationship between GDP and pharmaceutical spending is intertemporal in nature. We therefore assess whether there is any effect of lagged GDP on current pharmaceutical expenditure and that of lagged pharmaceutical expenditure on current GDP. We show results for up to three lags of GDP and pharmaceutical expenditure in Table 5. 10We lose almost 30% of our sample by the fourth lag of pharmaceutical expenditure. Hence, due to data limitations, the analysis is restricted to three lags. Although the point estimates of the effect of GDP per capita on pharmaceutical expenditure are quantitatively similar, they are statistically significant at a 10% level. Pharmaceutical expenditure, however, continues to show similar effects on GDP as before, both quantitatively and qualitatively. We discuss the negative effect of pharmaceutical spending on GDP in more detail in the conclusion section and provide several explanations in support of our findings.

**Table 5:** Intertemporal effects of GDP per capita and public pharmaceutical expenditure

Panel A: Lagged effect of GDP per capita on public pharmaceutical expenditures			
Variables	(1)	(2)	(3)
	Lag 1 year	Lag 2 years	Lag 3 years
GDP per capita (natural log)	1.856* (1.073)	2.182* (1.126)	2.083* (1.184)

Panel B: Lagged effect of public pharmaceutical expenditures on GDP per capita			
Variables	(1)	(2)	(3)
	Lag 1 year	Lag 2 years	Lag 3 years
Pharmaceutical exp.	−0.147*** (0.0415)	−0.143*** (0.0373)	−0.136*** (0.0361)
Year FE	Yes	Yes	Yes
Country FE	Yes	Yes	Yes
Observations	1,056	960	864
No. of countries	96	96	96

*Note*. Panel A (columns [1] to [3]) shows the lagged effect of GDP per capita (natural log) on public pharmaceutical expenditure using 1‐year to 3‐year lags of GDP per capita. Panel B shows the reverse effect, that is, of public pharmaceutical expenditure on GDP per capita using 1‐year to 3‐year lags of public pharmaceutical expenditure. All models include country and year fixed effects and a balanced sample of countries. GDP: gross domestic product.

*** *p* < 0.01.

** *p* < 0.05.

* *p* < 0.1.

So far, our results are consistent regarding the different specifications and sensitivity tests. However, concerns around the validity of the instrument may remain. We address such concerns in the next section related to instrument validity.

## 5 INSTRUMENT VALIDITY

The first stage F‐statistics clearly support the notion of relevance of the instrument. However, validity (exclusion restriction) is not directly testable. It is therefore worthwhile to discuss issues related to validity of the instrument and potential threats to validity. We show unadjusted and adjusted (partial) correlation coefficients in Tables A2a and A2b. When we look at the partial correlations, we find a strong relationship between GDP per capita and pharmaceutical expenditure. However, the partial correlation between tourist receipts and pharmaceutical expenditure is virtually zero; these descriptive findings therefore support the relevance and validity arguments for our instrument. Further, although it seems plausible to us that tourist receipts (spending by international visitors on food, travel, etc.) should not be related to public pharmaceutical expenditures of the host country directly other than through GDP, one can argue that medical tourism could be a potential source of contamination. Nevertheless, we argue that even if this were a likely source of correlation between the instrument and pharmaceutical spending, it seems plausible to presume that this spending will not be reflected in the public pharmaceutical expenditure of a country. In addition, it may also be argued that tourism itself may depend on the underlying health conditions of a country. For instance, an outbreak of a disease may affect the number of tourists visiting a country and therefore be related to GDP, health, and health expenditure. A priori one can expect disease outbreaks to be positively related to pharmaceutical expenditures (as they are likely to increase utilization of health services) and negatively related to GDP. In this scenario, if anything, our results would be an underestimate of the true effect. As unlikely as we perceive these effects to be on average, we collect data on disease outbreaks from 1996 up to the years in our sample. 11The data for disease outbreaks per country per year are from Google's public database on infectious disease outbreaks taken from HealthMap.org, Harvard Medical School. Due to missing data on most of the countries, we have a smaller sample in comparison with our main results. The data are available from https://www.google.com/publicdata/explore?ds=gh9ira29bjcpl_&ctype=l&strail=false&nselm=h&met_y=num_outbreaks&scale_y=lin&ind_y=false&rdim=whoregion&idim=whoregion%3AAfrica%3AAmericas%3AEastern+Mediterranean%3AEurope%3ASouth‐East+Asia%3AWestern+Pacific&tdim=true&tstart=&hl=en&dl=en [Last accessed February 23, 2018]. We first show in column (1) of Table 6 that there is no systematic relationship between disease outbreaks in a country and tourist receipts earned by the country, indicating that disease outbreaks are unlikely to affect tourism in our sample and thereby contaminate our IV strategy. Then, in column (2), we show the IV estimate of the effect of GDP per capita on public pharmaceutical expenditure per capita controlling for disease outbreaks. Our point estimates are robust to the inclusion of this control variable. Column (3) shows the effect of pharmaceutical expenditure on GDP controlling for disease outbreaks. We continue to find a negative and significant (at the 10% level) effect of pharmaceutical spending on GDP.

**Table 6:** Instrument validity

Variables	(1)	(2)	(3)
	Tourist receipts	Pharmaceutical expenditure	GDP per capita (natural log)
No. of outbreaks	−0.0500 (0.0353)	−0.0183 (0.0272)	0.0153 (0.0107)
GDP per capita (natural log)		2.251*** (0.484)	
Pharmaceutical exp.			−0.0739* (0.0431)
Year FE	Yes	Yes	Yes
Country FE	Yes	Yes	Yes
Observations	797	592	797
R‐squared	0.343	0.182	−0.213
No. of countries	71	65	71

*Note*. Column (1) shows the relationship between disease outbreaks in a country and tourist receipts. Column (2) shows the IV estimates of the effect of GDP per capita (natural log) on public pharmaceutical expenditure per capita controlling for disease outbreaks. Column (3) shows the IV estimates of the effect of public pharmaceutical expenditure per capita on GDP per capita (natural log) controlling for disease outbreaks. GDP: gross domestic product; IV: instrumental variable.

*** *p* < 0.01.

** *p* < 0.05.

* *p* < 0.1.

## 6 EFFECT HETEROGENEITY

The fixed effects in our analysis ensure that long‐standing differences between countries do not bias the estimates in any way, that is, differences pertaining to the country level such as preferences for spending on health care and production functions play no role. However, it is likely that the elasticity of pharmaceutical expenditure differs across countries as is observed in past literature on health expenditures. Prior literature that investigates elasticity of health expenditure usually assesses heterogeneity by simply segmenting countries into income groups, development levels, health spending, and time periods (Blazquez‐Fernandez, Cantarero, & Perez, 2014; Farag et al., 2012). We extend the investigation of effect heterogeneity in several ways by considering (a) different quantiles of the response variables and (b) politico‐economic freedom of countries. 12We are grateful to a reviewer for recommending these strategies that have helped enrich the analysis.


### 6.1 Income groups

First, similar to prior research on determinants of health expenditure, we investigate heterogeneity by income group. Columns (1) and (3) of Table 7.1 show the estimates of the effect of GDP per capita on public pharmaceutical expenditure for high‐ and low‐income countries, respectively. The effect is significant only for the group of high‐income countries where the elasticity exceeds unity; however, it is not significant for low‐income countries. Columns (2) and (4) show the corresponding estimates of the effect of public pharmaceutical expenditure on GDP per capita. The estimates are negative, significant at a 5% level only for low‐income countries and not significant at any conventional level for high‐income countries. It is important to note here that due to sample size issues, we classified high‐income and upper middle‐income countries together as high‐income countries. Similarly, we classified low‐income and lower middle‐income countries together as low‐income countries. We therefore need to bear in mind that not all high‐income countries and not all low‐income countries are at the same level of GDP or pharmaceutical spending. For example, in our sample in 2006, the difference between GDP per capita of the country with the highest GDP (Luxembourg) in the high‐income group and the country with the lowest GDP (Fiji) in the upper middle‐income group was as much as U.S. $99,328. Similarly, the difference is as much as U.S. $5,345 between the country with the highest GDP (Colombia) in the lower‐middle income group and the lowest GDP country (Ethiopia) in the low‐income group. There is high variation in both GDP and pharmaceutical expenditure within the two income groups and therefore combining these countries in the same group may not provide an accurate picture. We therefore employ additional strategies to assess heterogeneity, as described next.

**Table 7.1:** Effect heterogeneity: Income groups

	High income		Low income	
	(1)	(2)	(3)	(4)
Variables	Pharmaceutical expenditure	GDP per capita (natural log)	Pharmaceutical expenditure	GDP per capita (natural log)
GDP per capita (natural log)	1.766*** (0.849)		1.319 (1.017)	
Pharmaceutical exp.		−0.136 (0.092)		−0.098*** (0.038)
Year FE	Yes	Yes	Yes	Yes
Country FE	Yes	Yes	Yes	Yes
Observations	797	797	592	592
R‐squared	0.531	0.445	0.315	0.481
First stage F‐stat	24.81	56.55	4.28	474.10
*p* value	0.000	0.000	0.042	0.000
Kleibergen‐Paap LM statistic	6.58	15.35	3.66	15.37
*p* value	0.0103	0.0001	0.0557	0.0001
Endogeneity test	2.221	4.586	2.081	10.677
*p* value	0.1362	0.0322	0.1491	0.0011
Number of countries	71	71	65	65

*Note*. The DV in columns (1) and (3) is public pharmaceutical expenditure per capita; in columns (2) and (4), it is GDP per capita (natural log). Columns (1) and (2) show IV estimates for high‐income countries whereas columns (3) and (4) show IV estimates for low‐income countries. GDP: gross domestic product; IV: instrumental variable.

*** *p* < 0.01.

** *p* < 0.05.

* *p* < 0.1.

### 6.2 IV quantile regressions

We recognize the importance of going beyond conditional means, something that the empirical economics literature makes a compelling case for (Koenker & Hallock, 2001), and adopt an IV quantile regression approach to assess the effects of GDP and pharmaceutical expenditure. Quantile regressions are robust to nonnormal errors and outliers. In addition, they allow us to estimate the impact of GDP per capita on the entire distribution of pharmaceutical expenditure and vice versa.

Table 7.2 shows the results of the IV quantile regressions. Panel A shows the results of the income elasticity of pharmaceutical expenditure at the 10th, 25th, 50th, 75th, and 90th quantiles of pharmaceutical expenditure. We find that the elasticity systematically decreases as we move to higher quantiles of expenditure. For countries that are at the lower end of the distribution, that is, spend very little on pharmaceutical expenditure, a 1% increase in GDP per capita leads to an approximately 2% increase in pharmaceutical expenditures. However, for countries at the upper end of the distribution we find that the increase in pharmaceutical expenditure in response to an increase in GDP per capita is near unity. The results seem plausible given that countries with limited spending on pharmaceuticals increase spending with increases in GDP, because they have not yet reached the “flat part of the curve” of medical spending, that is, marginal productivity of expenditure may still be increasing (Farag et al., 2012). However, countries that are already at the high end of spending face diminishing returns to health spending and, hence, contain health spending.

**Table 7.2:** Effect heterogeneity: Quantile IV regressions

Panel A: Effect of GDP per capita on public pharmaceutical expenditures					
	(10th)	(25th)	(50th)	(75th)	(90th)
Variables					
GDP per capita (natural log)	1.957*** (0.286)	1.320*** (0.153)	1.205*** (0.121)	1.056*** (0.0781)	1.069*** (0.0565)

Panel B: Effect of public pharmaceutical expenditures on GDP per capita					
	(10th)	(25th)	(50th)	(75th)	(90th)
Variables					
Pharmaceutical exp.	−0.0290 (0.0206)	−0.0486*** (0.00772)	−0.0490*** (0.0121)	−0.0509*** (0.0158)	−0.0398 (0.0265)
Year FE	Yes	Yes	Yes	Yes	Yes
Country FE	Yes	Yes	Yes	Yes	Yes
Observations	1,389	1,389	1,389	1,389	1,389
No. of countries	136	136	136	136	136

*Note*. The DV in panel A is public pharmaceutical expenditure per capita, and in panel B, it is the natural log of GDP per capita. The coefficients represent estimates from IV quantile regressions at the 10th, 25th, 50th, 75th, and 90th quantile of the respective dependent variable as indicated. All regressions include year fixed effects and country fixed effects. GDP: gross domestic product; IV: instrumental variable.

*** *p* < 0.01.

** *p* < 0.05.

* *p* < 0.1.

The difference in the income elasticity of pharmaceutical expenditure between an analysis by pharmaceutical expenditure (quantile regressions) and an analysis by income is noteworthy because countries spending less on pharmaceuticals are also poorer countries; Table A3 in the appendix shows the percentage of high‐ and low‐income countries at each quantile of pharmaceutical spending. However, there is significant between‐country heterogeneity even within an income group. For example, between‐country variation in GDP per capita in the low‐income group is as much as $1,234.63. This heterogeneity between countries reduces to half for low‐income countries with low spending, that is, lowest quantile of pharmaceutical expenditure, thus pointing towards the role of spending. Therefore, a simple classification into high‐ and low‐income groups does not show the complete picture; quantile regressions enrich our understanding of the results and reveal that the existing spending level is important for explaining heterogeneity. Although prior literature that analyses overall health expenditures finds an income elasticity of less than unity over the entire distribution (Tian, Gao, & Yang, 2016), results for specific type of expenditure such as pharmaceuticals thus show a different picture.

With respect to the impact of pharmaceutical spending on GDP per capita, we find that the results are negative and significant only for the 25th, 50th, and 75th quantiles. These results imply that pharmaceutical expenditure does not have any impact on GDP at the lowest and the highest end of the distribution, that is, in the poorest and richest countries. These results are to some extent similar to the results we observe with the income groups where the richer countries do not show a significantly negative effect of pharmaceutical spending. We posit that the budget constraint these countries face relative to middle‐ and low‐income countries is not as stringent in that, they invest significantly in other productive types of government activities as well. As such, the investment diversion that low‐income countries face might be higher.

### 6.3 Politico‐economic situation

As the World Health Report, 2000, argues, health law enforcement and political‐economic conditions play an important role in shaping national policies on health care that may be either health promoting or health restricting. For example, trade embargoes in countries can restrict import–export of essential medicines, thereby affecting health outcomes. Hence, the degree of political freedom in a country may determine health policies and subsequent health spending. As such, two countries may grow apart by their income levels; yet it is not income but political and economic freedom that determines spending on pharmaceuticals. 13We are grateful to a referee for pointing this out.


We therefore assess heterogeneity in the effects by political‐economic situation (see Table 7.3) using the EFW index from the Fraser Institute. 14Data are available openly from https://www.fraserinstitute.org/economic‐freedom/dataset?min‐year=1970&max‐year=2015&filter=0&date‐type=range&geozone=world&page=dataset. For details on the EFW index, we refer readers to https://www.fraserinstitute.org/. We use data on the overall score along with scores on civil and political rights that classify countries as economically “free” or “not free.” We find that GDP per capita has a significant impact on pharmaceutical spending only in countries classified as “free.” It is not significant for countries classified as “not free.” This implies that for these countries, it is not GDP, but the political environment that matters. In contrast, we find that the impact of pharmaceutical expenditure has a significantly negative effect only in countries that are “not free.” It is not significant for countries classified as “free.”

**Table 7.3:** Effect heterogeneity: Politico‐economic freedom

Panel A: Effect of GDP per capita on public pharmaceutical expenditures						
	Civil liberty		Political rights		Overall score	
	(1)	(2)	(3)	(4)	(5)	(6)
	Free	Not free	Free	Not free	Free	Not free
GDP per capita (natural log)	1.573*** (0.603)	1.486 (0.937)	1.854*** (0.712)	1.606 (1.212)	1.479** (0.619)	1.361 (0.972)

Panel B: Effect of public pharmaceutical expenditures on GDP per capita						
	Civil liberty		Political rights		Overall score	
	(1)	(2)	(3)	(4)	(5)	(6)
	Free	Not free	Free	Not free	Free	Not free
Pharmaceutical exp.	−0.0292 (0.0260)	−0.157** (0.0660)	−0.0461 (0.0296)	−0.192** (0.0837)	−0.00344 (0.0232)	−0.151** (0.0636)
Year FE	Yes	Yes	Yes	Yes	Yes	Yes
Country FE	Yes	Yes	Yes	Yes	Yes	Yes
Observations	654	733	791	591	780	601
No. of countries	66	89	79	78	78	79

*Note*. The DV in panel A is Public pharmaceutical expenditures, and in Panel B, it is GDP per capita. The EFW index is used to classify countries as “free” and “not free” based on civil liberties (columns [1] and [2]), political rights (columns [3] and [4]), and an overall score (columns [5] and [6]). All regressions include year fixed effects and country fixed effects. DV: dependent variable; EFW: Economic Freedom of the World; GDP: gross domestic product; IV: instrumental variable.

*** *p* < 0.01.

** *p* < 0.05.

* *p* < 0.10.

Overall, heterogeneity of countries with respect to economic development, pharmaceutical spending, and political environment plays an important role. As the results show, low‐income countries respond differently to an increase in GDP and an increase in health spending compared with high‐income countries. Most of the low‐income countries in our sample are also classified as “not free” (75%), whereas most of the high‐income countries are classified as “free” (80%). This heterogeneity in the effects can have important implications for pharmaceutical policy and regulation, which we discuss in Section 8.

## 7 EXPLAINING THE NEGATIVE EFFECT OF PUBLIC PHARMACEUTICAL EXPENDITURE

An obvious question that arises from the results concerns the negative effect of public pharmaceutical expenditure on GDP per capita, that is, how the negative effect can be explained. Theoretically, Ram (1986) suggests that government size can be growth reducing; empirically, findings of some of the prior literature support this notion (Barro, 1991; Landau, 1983). We draw on this literature and provide arguments that are both general to government spending and specific to pharmaceutical spending. Government size can be harmful for growth due to inefficiency, regulatory costs, and distorted economic incentives arising out of government fiscal policies (Ram, 1986). Distortionary taxes to finance government spending may negatively affect savings and hence growth (Barro, 1991). If public spending crowds out private investment, and if government spending is on inefficient and unproductive activities, it can impinge negatively on growth (Afonso & Furceri, 2010). Financing of government spending by means of increased taxation may have negative spillovers to the economy (Asimakopoulos & Karavias, 2016).

Another reason through which public spending on pharmaceuticals affects GDP negatively may be an investment diversion effect. With respect to health spending, others (Acemoglu & Johnson, 2007; Ashraf et al., 2008) have found that it takes a long time for health‐related economic returns to be witnessed. Even if we witness health benefits that translate into economic returns contemporaneously, they might not be large enough to justify the disproportionate spending, which is an opportunity cost in itself and diverts resources away from potentially more productive sources of public expenditure such as transport and infrastructure spending (Easterly & Rebelo, 1993; Kelley, 1988). Third, most countries have a significant demand for imported pharmaceutical products, and spending a lot on imported drugs may lead to a downward multiplier effect in the economy. For example, in the Unites States, direct imports of pharmaceuticals rose from $15.4 billion out of the $63.3 billion pharmaceutical sales in 1998 to around $68 billion out of the $151 billion sales in 2012 (Werling et al., 2014). Finally, the method of financing government expenditures might play a crucial role in determining the relationship between spending and growth; debt financed increases in health spending can have negative effects on economic growth (Ahmed & Miller, 1999). Increased government borrowing and higher taxes to finance health expenditures can be detrimental to economic growth in that it reduces income for private spending. A large part of health expenditure is indeed financed by taxes or social insurance contributions (Gerdtham & Jönsson, 2000). In addition, spending on ineffective drugs (in terms of both cost and illness) results in unproductive expenditure, which does not contribute to the economy. This may be particularly true for less developed and politico‐economically unfree countries that not only lack efficient allocative mechanisms but also face accessibility problems to high quality medicines (Kyle & McGahan, 2012). Our results are particularly significantly negative for such less developed and poor countries. Pharmaceuticals covered by public resources in these countries may not be cost‐saving or even cost‐effective because public resources may be misdirected. Hence, the allocation of government expenditures may not be rational in this sense. As a result, inefficient spending coupled with a stringent budget constraint raises the opportunity cost and reduces productive returns much more in these countries. Although we are unable to investigate specifically each mechanism due to data unavailability, we leave this avenue open for future research. We discuss the implications of our findings for policy in the next section.

## 8 CONCLUSION

This paper answers two questions that are both important and strongly debated in the health economics literature. First, it assesses the income elasticity of medical spending focusing on public pharmaceutical expenditures. Second, it assesses the effect of public pharmaceutical spending on income measured by GDP per capita. To this end, the paper employs a two‐step IV procedure using data for 136 countries from 1995 to 2006 and answers both research questions within the same econometric framework. Briefly, our IV estimates suggest that the least squares regression model significantly underestimates the response of public pharmaceutical spending to GDP per capita, and once endogeneity is accounted for, public pharmaceutical expenditure shows an income elasticity that is greater than unity. Further, once the reverse causal effect of GDP on pharmaceutical spending is accounted for, public pharmaceutical spending itself has a negative effect on GDP per capita. Heterogeneity of countries with respect to economic development, pharmaceutical spending, and politico‐economic environment plays an important role in the understanding of these results and their implications for health policy. Overall, countries that spend little on pharmaceuticals show an income elasticity above one, whereas countries that spend a lot are unitary elastic. Countries that are politico‐economically “free” in our analysis show elasticity greater than unity, whereas countries that are “not free” show no significant relationship with income. On the contrary, pharmaceutical spending itself has a negative impact on GDP in “unfree” countries and countries at the lower end of the distribution of GDP.

Given these results, the question is how do they inform health care policy. As Acemoglu et al. (2013) point out, understanding the role of income in the rise of health expenditures is important for forecasting the evolution of health expenditures and analyzing the social optimality of health spending. Further, an assessment of the elasticity of pharmaceutical spending is crucial for policy in that it will aid policy makers in deciding whether to intervene in the market or to let market forces play a role in improving welfare. Finally, our findings show that countries that spend little on pharmaceuticals have an income elasticity greater than one; which begs the question—are we achieving convergence in pharmaceutical spending across the world. 15Note that Farag et al. (2012) find lower income elasticity of health spending and argue to the contrary that convergence in health and health spending is unlikely anytime soon. Concerning the negative effect of pharmaceutical spending on GDP, the results point towards a need for improving efficiency and effectiveness of medical spending as well as limiting waste. Although “irrational” use of medicines is a problem in both low‐ and high‐income countries, in developed countries, routine monitoring and strong electronic systems limit wasteful spending to a certain extent. In developing countries, however, such monitoring systems are not available, resulting in more than 60% of the patients in the public sector not being treated according to appropriate guidelines (WHO, 2011). Furthermore, even if medicines are prescribed appropriately, patient adherence is weak with over 80% drugs being dispensed by unqualified personnel and about one‐third of patients lacking knowledge on how to take medicines (WHO, 2011). Therefore, despite incurring costs, medicines may not translate into health benefits.

Furthermore, high‐income countries have strong regulations such as reference pricing, national or regional procurement contracts and reimbursement policies that provide them with a certain degree of control over prices. As a result, there is less differential between originator brands and lowest price generics than in low‐income countries where the difference may be over 300% (WHO, 2011). Therefore, the higher prices that low‐income countries are subjected to further raise the opportunity cost of spending on pharmaceuticals.

Improving allocative efficiency of government budgets taking into account the opportunity costs of spending on health care, and investing optimally in other areas are crucial steps for the sustainability of fiscal budgets. An important policy implication due to the heterogeneity in the results is that encouraging the uptake of cost‐effective medicines in less developed countries may be key for efficient health spending. Revill et al. (2014) argue that current cost‐effectiveness thresholds set by the WHO are not adequate as they impose opportunity costs under budget constraints. Given our findings of the negative effect on growth, this becomes important for long‐term sustainability of health care systems in these countries. On a similar note, policy should be geared towards price control so that more can be achieved with the same level of spending without having to reduce volume (which is not an option in low‐income countries anyway given the low level of spending).

The results based on the politico‐economic freedom mean that greater politico‐economic freedom may help counter the negative effect of health spending by improving within‐ and cross‐border pharmaceutical trade, thus increasing access to essential medicines. This may improve the marginal productivity of health expenditure. Also, providing subsidies and encouraging local R&D to reduce dependence on imported and expensive pharmaceuticals may help counter the negative multiplier effect in the economy. Finally, it needs to be acknowledged that even if economic growth is negative and pharmaceutical spending is wasteful to some degree, there may be reasons beyond economic growth to increase public funds for pharmaceuticals, for example, a reduction in inequality in access to pharmaceuticals.

Before we conclude, it is important to mention a few caveats of the analysis and provide recommendations for future research related to this topic. First, our analysis largely addresses contemporaneous effects. The short‐term perspective is of particular interest to policy makers as they generally have a short planning horizon. This is similar to the perspective of a budget impact analysis, a standard tool for estimating the financial consequences of adopting new health interventions. However, long‐run effects cannot be ruled out, and hence, we analyze lagged effects within the limits of data availability. As longer time series data becomes available, future work should look into long‐run elasticities of health spending and the long‐run effect of health spending on income. As a word of caution, even in the long‐term increased public pharmaceutical expenditure may not lead to economic growth because of the reasons mentioned previously and because savings in health expenditures may not be realized (see, e.g., Shroufi et al., 2013, and Horton et al., 2017). Second, we consider public pharmaceutical spending because it forms a major part of total health expenditure and is exposed to many regulations. Needless to say, that future work should consider different types of health spending such as preventive and rehabilitative expenditures. Finally, we consider pharmaceutical expenditure in its final form ignoring that it is a combination of price and quantity (Lu, Hernandez, Abegunde, & Edejer, 2011). To the extent that prices reflect the innovativeness of a drug or a drug being branded/generic, and quantity reflects the disease burden of the country, analyzing the elasticity by separating expenditures into these components could provide interesting insights for policy. As better data become available in the future, this could be a potential question for researchers to pursue.

## CONFLICTS OF INTEREST

None

## FUNDING SOURCES

This research did not receive any specific grant from funding agencies in the public, commercial, or not‐for‐profit sectors.

## APPENDIX A

### Appendix A.1 xxx

**Table A1:** Countries and number of observations per country

Country	Observations	Country	Observations	Country	Observations
Albania	4	Fiji	12	Netherlands	12
Algeria	12	Finland	12	New Zealand	12
Antigua and Barbuda	12	France	12	Nicaragua	12
Argentina	12	Gabon	11	Niger	2
Armenia	12	Georgia	10	Norway	12
Australia	12	Germany	12	Oman	2
Austria	12	Greece	12	Pakistan	7
Azerbaijan	12	Grenada	12	Panama	12
The Bahamas	12	Guatemala	12	Papua New Guinea	12
Bahrain	12	Guinea	2	Paraguay	12
Bangladesh	12	Guyana	12	Peru	12
Barbados	12	Haiti	11	Philippines	12
Belarus	12	Honduras	12	Poland	12
Belgium	12	Hungary	12	Portugal	12
Belize	12	Iceland	12	Russian Federation	12
Benin	2	India	12	Rwanda	2
Bhutan	10	Indonesia	12	Samoa	12
Bolivia	12	Ireland	12	Senegal	12
Bosnia and Herzegovina	4	Israel	12	Serbia	5
Brazil	12	Italy	12	Singapore	7
Brunei Darussalam	6	Jamaica	12	Slovak Republic	12
Bulgaria	12	Japan	12	Slovenia	12
Burkina Faso	7	Jordan	3	Solomon Islands	8
Cabo Verde	12	Kazakhstan	12	Spain	12
Cambodia	12	Kenya	7	Sri Lanka	12
Cameroon	7	Kiribati	8	St. Kitts and Nevis	12
Canada	12	Korea, Rep.	12	St. Lucia	12
Central African Republic	12	Kuwait	2	St. Vincent and the Grenadines	12
Chad	8	Kyrgyz Republic	12	Suriname	12
Chile	12	Lao PDR	12	Sweden	12
China	12	Latvia	12	Switzerland	12
Colombia	12	Lesotho	3	Thailand	12
Costa Rica	12	Lithuania	12	Tonga	10
Croatia	12	Luxembourg	12	Trinidad and Tobago	12
Cyprus	12	Macedonia, FYR	12	Tunisia	4
Czech Republic	12	Malawi	4	Turkey	9
Denmark	12	Malaysia	12	Uganda	8
Djibouti	7	Maldives	6	Ukraine	12
Dominica	12	Mali	7	United Kingdom	12
Dominican Republic	12	Malta	12	United States	12
Ecuador	12	Mexico	12	Uruguay	12
El Salvador	12	Moldova	10	Uzbekistan	7
Equatorial Guinea	7	Mongolia	12	Venezuela, RB	4
Estonia	12	Myanmar	12	Vietnam	4
Ethiopia	2	Nepal	12	Yemen, Rep.	3
Zambia	7				

**Table A2a:** Pearson's correlation coefficients between public pharmaceutical expenditure, GDP per capita, and tourist receipts

	Public pharmaceutical expenditure	GDP per capita (natural log)
GDP per capita (natural log)	0.9193	
*p* value	(0.0000)	
Tourist receipts	0.6027	0.6751
*p* value	(0.0000)	(0.0000)

*Note*. The table shows Pearson's correlations. The *p* value is reported in parentheses below the correlation coefficient. GDP: gross domestic product.

**Table A2b:** Partial correlations between public pharmaceutical expenditure, GDP per capita, and tourist receipts

	Public pharmaceutical expenditure	GDP per capita (natural log)
GDP per capita (natural log)	0.8704	—
*p* value	(0.0000)	—
Tourist receipts	−0.0616	0.3854
*p* value	(0.0217)	(0.0000)

*Note*. The table shows partial correlations. The *p* value is reported in parentheses below the correlation coefficient. GDP: gross domestic product.

**Table A3:** Percentage of high‐ and low‐income group of countries at quantiles of pharmaceutical expenditure

Quantiles of public pharmaceutical expenditure					
	1 (%)	2 (%)	3 (%)	4 (%)	5 (%)
High‐income group	2.13	29.03	60.79	96.77	100.00
Low‐income group	97.87	70.97	39.21	3.23	0.00
